# Encoding information into autonomously bursting neural network with pairs of time-delayed pulses

**DOI:** 10.1038/s41598-018-37915-7

**Published:** 2019-02-04

**Authors:** June Hoan Kim, Ho Jun Lee, Wonshik Choi, Kyoung J. Lee

**Affiliations:** 1 Department of Physics, Korea University, Seoul, 02841 Korea; 2 Center for Molecular Spectroscopy and Dynamics, Institute for Basic Science, Seoul, 02841 Korea

## Abstract

Biological neural networks with many plastic synaptic connections can store external input information in the map of synaptic weights as a form of unsupervised learning. However, the same neural network often produces dramatic reverberating events in which many neurons fire almost simultaneously – a phenomenon coined as ‘population burst.’ The autonomous bursting activity is a consequence of the delicate balance between recurrent excitation and self-inhibition; as such, any periodic sequences of burst-generating stimuli delivered even at a low frequency (~1 Hz) can easily suppress the entire network connectivity. Here we demonstrate that ‘Δ*t* paired-pulse stimulation’, can be a novel way for encoding spatially-distributed high-frequency (~10 Hz) information into such a system without causing a complete suppression. The encoded memory can be probed simply by delivering multiple probing pulses and then estimating the precision of the arrival times of the subsequent evoked recurrent bursts.

## Introduction

Neural networks can generate population bursts (PBs), which are an almost simultaneous firings of many neurons comprising the system. They are a very significant nonlinear dynamic feature of the brain, often being associated with neuro-pathological states like epilepsy^1^, Parkinson’s disease^2^ or schizophrenia^3^. As for treating these diseases, they need to be avoided at all cost. On the other hand, PBs do play essential roles in various fundamental neural processes of sensory networks^4,5^, pattern-generating circuits^6^, thalamocortical circuits^7^, and in maintaining neural memory^8^. Thus it is imperative to understand the underlying biophysical mechanisms responsible for the generation of PBs and explore how their spatiotemporal dynamics is involved in neural information processing.

Autonomous PB dynamics supported by the brain are in many cases very erratic showing no discernible spatiotemporal patterns^9–11^. Equally complex PB dynamics can be generated by high-density dissociated neuronal cell cultures^12,13^. In both cases the complexity of the PB temporal sequences is often attributed to various ‘neuronal noise’ present in the system^14–16^. But, at least in part the complexity stems at some deterministic factors, as several careful analyses show that PBs can recur repeatedly with specific motifs^17–19^ and as seemingly random sequence of PB events can be attributed to the existence of several (hidden) sub/supra-threshold ‘PB oscillators’^13,20^.

In order to understand neuronal PB dynamics and its role in neural information processing more actively, several experimental studies investigated the effects of various extrinsic electrical perturbations. Different modes of stimulation have been explored and tested, and the subsequent stimulation effects were evaluated, by quantifying either the changes in the spontaneous PB dynamics itself^21–25^ or the response of the system following a probing pulse^26–29^. Furthermore, Wagenaar *et al*.^22^ demonstrated a feedback control of PB intervals and their ‘burstiness’ using a quite complex spatiotemporal stimulation protocol. Also, Chiappalone *et al*.^28^ introduced a stimulation pattern that was modeled after an actual sequence of PBs and its effectiveness was discussed with respect to the phenomenon of long term potentiation (LTP). However, none of these studies clearly explained the underlying mechanisms responsible for their experimental results.

Recently, we introduced a unique stimulation protocol which is now coined as ‘Δ*t* paired-pulse stimulation’ as for training PB generating neuronal cell cultures^29^. The stimulation delivers a long periodic sequence (typically, 200 times at 5 s interval) of bipolar pulses (400 mV amplitude, 250 *μ*s duration for each phase) to two selected groups of electrodes (neurons) with a relative time delay of Δ*t* (typically, ranging −30~30 ms) between the two groups^29^. It was shown that the Δ*t* stimulation protocol was very effective in changing the ‘overall excitability’ of neuronal cell cultures, which qualitatively represents the likelihood of a PB generation. Incidently, a similar stimulation protocol termed as “desynchronizing delayed feedback stimulation” is found to be very effective in the treatment of several neurological disorders like Parkinson’s disease (see^30,31^ and the references therein). In this paper, we explain how the particular stimulation protocol could have worked for the system and discuss its novelty in connection with a synaptic memory formation in a PB supporting neuronal system. A detailed explanation is given based on computer simulations of the well-known Izhikevich neuronal network model^32^, which is well-known for its population bursting dynamics. A few years ago, we demonstrated that the Izhikevich model supports the phenomenon of coherence resonance when it is driven by different levels of noise: in other words, there is an optimal level of noise at which the variation of the interburst interval becomes minimal^13^. In this study, we use the same model for a very different purpose, specifically, to explore the role of Δ*t* paired-pulse stimulation as for rendering a meaningful change in the network synaptic morphology and the related PB dynamics. Here, the interplay among the value of Δ*t*, the duration of PB, and the time relevant for the spike-timing-dependent-plasticity (STDP) mechanism, which is built into the model system, is found to be essential.

## Mathematical model of PB generating neuronal network

So far, several different mathematical models have been introduced to generate spontaneous neuronal PB oscillations^32–35^. In this study we use the Izhikevich neural network model^32,36^, since it is easy to simulate on a personal computer to generate some realistic PB dynamics^13^. More importantly, the model incorporates a STDP rule–an essential feature for synaptic memory formation and removal. Although the details are rather different, all previous mathematical models of PB generating networks include two competing mean-field factors–*activation* and *inhibition*. For the model proposed by Augustin *et al*.^35^, the two factors are recurrent synaptic excitation and neuronal adaptation (current), respectively. Similarly, the Izhikevich model^32,36^ includes recurrent excitatory (and fewer inhibitory) synaptic connections, while burst inhibition is indirectly implemented via the asymmetric STDP curve that is biased toward the suppression side over the potentiation side.

**Figure 1:**
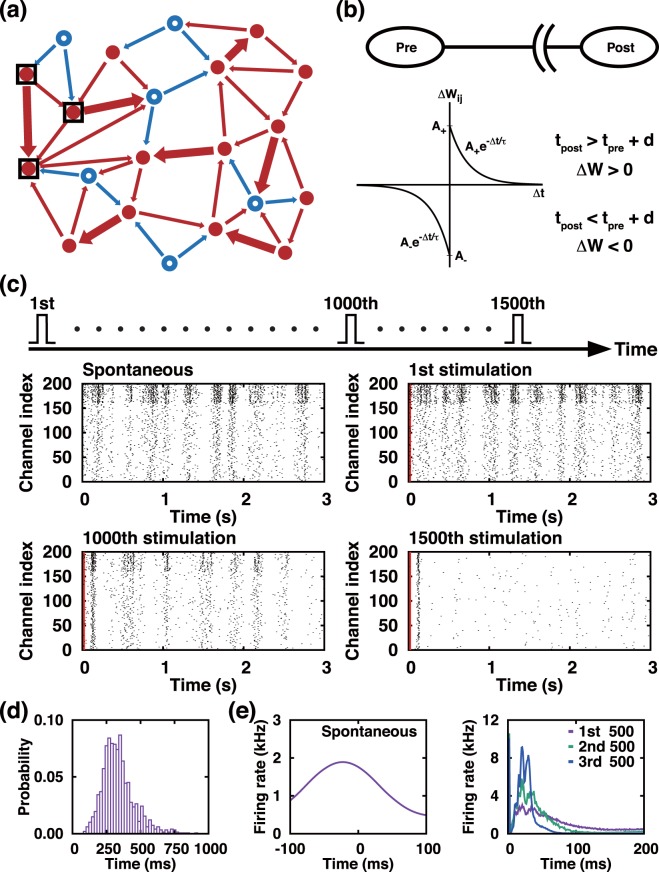
Progressive depression in bursting neural network by a periodic sequence of single-pulse stimuli. (**a**) Schematic illustration showing a randomly connected excitatory (red filled circles) and inhibitory (blue open circles) neurons. (**b**) Skewed STDP function of plastic synapses, (**c**) Raster plots of APs changing with the number of externally delivered stimuli [first frame: autonomous activity just before the stimulation starts, other frames: AP activities following each stimulation (vertical red line at *t* = 0 marks the time of the stimulation). The same pulse is delivered repetitively at every 3 s interval to a subset (20 neurons) of the population. (**d**) Histogram of 500 successive inter-burst-intervals of an autonomously generated PB sequence. (**e**) AP firing rate profile of autonomously generated PB (left frame) and those of evoked PBs immediately following the stimulating pulse (right frame) [All are an average profile based on 500 PBs]. The red (blue) arrows in (**a**) represent the direction of AP propagation and the strength of connections. In (**b**), *t*_*pre*_ (*t*_*post*_) and *d* represent, the spiking time of presynaptic (postsynaptic) neuron and conduction delay, respectively. In the raster plots of (**c**), the top 40 (bottom 160) lines are for the inhibitory (excitatory) neurons.

Specifically, our model is comprised of 200 (160 excitatory and 40 inhibitory) integrate-fire neurons coupled via a STDP function as described in Fig. 1b. The state of individual neuron evolves according to this set of equations:

1 $$C\frac{dv}{dt}=k(v-{v}_{r})(v-{v}_{t})-u+I$$

2 $$\frac{du}{dt}=a(bv-u).$$

As the value of *v* crosses a prescribed threshold value (30.0 mV), the two variables are made to reset:

$${\rm{if}}\,{v}+{\rm{30.0}}\,{\rm{mV}},\,{\rm{then}}\,\{\begin{array}{ccl}v & \leftarrow  & {\rm{c}}\\ u & \leftarrow  & u+d.\end{array}$$

In the above model, the variables, *v* and *u*, are the membrane potential and the level of recovery following a reset (i.e., spike firing), respectively. *I* is the sum of synaptic current inputs (*I*_*syn*_) from other neurons, an external current (*I*_*ext*_) and a residual noisy current (*I*_*η*_). *I*_*η*_ = 16 pA was given to only one randomly chosen neuron at every 1 ms. The variable external current was used only for the Δ*t* training or for inducing recurrent PBs for evaluating the time precision of recurrent PBs; otherwise, it was set to be zero. Throughout this paper, the following parameter values are used: *v*_*r*_ = −82.7 mV, *v*_*t*_ = −42.3 mV, *C* = 1 nF, *k* = 0.04 pA/mV^2^, *b* = 0.2 nS, *c* = −65 mV, and *a* = 0.02 (0.1) ms^−1^ and *d* = 8 (2) pA for excitatory (inhibitory) neurons.

The number of synaptic connections (to postsynaptic neurons) for each neuron is chosen to be 60. The inhibitory neurons are projected only to excitatory neurons with a fixed synaptic weight of −5 and a fixed conduction delay of 1 ms. On the other hand, the excitatory neurons project to inhibitory as well as excitatory neurons with time-varying (0~10) synaptic weights *W*_*ij*_, and with randomly (but uniformly) selected conduction time delays *τ*_*ij*_ (1~20 ms), which are what we have typically measured for the inter-channel conduction delays within cultured networks of neurons. The synaptic current to the *i*th neuron is:

$${I}_{i,syn}=\sum _{j}\sum _{m}{W}_{ij}\delta (t-({t}_{j}^{m}+{\tau }_{ij})),$$

in which ${t}_{j}^{m}$ is the time of *m*th spiking of *j*th neuron.

The weight *W*_*ij*_ (of a synapse connecting a presynaptic neuron *j* to a postsynaptic neuron *i*) of excitatory neurons is updated every second based on the STDP function depicted in Fig. 1b: The change in *W*_*ij*_ (Δ*W*_*ij*_) depends on the temporal distance Δ*t*_*ij*_ between the time *t*_*j*_ of presynaptic spike and the time *t*_*i*_ of postsynaptic spike. We define the sequence of presynaptic spiking times to be ${t}_{j}^{m}$ and that of postsynaptic spiking times to be ${t}_{i}^{n}$, where *m* and *n* = 1, 2, 3… are temporal indices for the action potentials in sequence. Incorporating the conduction time delays *τ*_*ij*_, the actual time difference between the m*th* spike of presynaptic neuron (*j*) and the n*th* spike of postsynaptic neuron (*i*) is ${\rm{\Delta }}{t}_{ij}^{nm}={t}_{i}^{n}-({t}_{j}^{m}+{\tau }_{ij})$. Thus, at each iteration time-step:

$${\rm{\Delta }}\,{W}_{ij}=\sum _{n}\sum _{m}W({\rm{\Delta }}{t}_{ij}^{nm}),$$

where

$$W({\rm{\Delta }}{t}_{ij}^{nm})={A}_{+}\,\exp (\,-\,{\rm{\Delta }}{t}_{ij}^{nm}/\tau )\,{\rm{for}}\,({\rm{\Delta }}{t}_{ij}^{nm}\, > \,0)\,{\rm{and}}\,-\,{A}_{-}\exp ({\rm{\Delta }}{t}_{ij}^{nm}/\tau )\,{\rm{for}}\,({\rm{\Delta }}{t}_{ij}^{nm}\, < \,0).$$

Throughout this paper, we used the parameter values of *A*_+_ = 0.1, *A*_−_ = 0.12, and *τ* = 20 ms. The choice of parameter *A*_−_ > *A*_+_ suppresses the potential over-excitation of the network. And, as we will explain shortly, the conduction time delay also plays a delicate role as for bringing inhibition to a bursting network.

Our numerical simulation was carried out by customizing the c^++^ code, which was originally published in^32^.

## Results

### Network bursting activity and its suppression by a periodic sequence of single-pulse stimuli

The raster plot in the top-left frame of Fig. 1c shows a typical PB event series (of 3 seconds) that is autonomously generated by our model network. The histogram of the inter burst intervals of spontaneously generated PBs in general shows a rather broad distribution about 300 ms as shown in Fig. 1d. In fact, earlier we suggested that the time series can be viewed as a very noisy PB oscillation^13^, which is formed by two competing factors: inhibition via the depression-favored STDP rule and recurrent synaptic excitation.

During each individual PB event, the cell-to-cell synaptic connections are to be weakened in general. There are two different elements in this weakening process. First of all, as mentioned previously the STDP function itself is biased towards the inhibition side: In the original model proposed by Izhikevich this bias was intentionally introduced to avoid network over-excitation^32^. Second, there is a conduction delay *τ*_*ij*_ (which is randomly chosen from 1 to 20 ms) of the presynaptic firing of *i*th neuron towards its *j*th postsynaptic neurons. With the delays, the synaptic connection weights *W*_*ij*_ are more likely to decrease than increase as each PB event comes to an end. This suppression effect is easy to understand if we imagine an unusual PB in which all cells fire simultaneously. For such a case, all synaptic weights will decrease because all post-synaptic neurons fire effectively before their presynaptic partners do (due to conduction delays). For autonomously generated PBs, cells do not fire simultaneously but their AP spikes are dispersed as shown in the left frame of Fig. 1e, more or less in a form of Gaussian function. Even these Gaussian PBs will cause an overall synaptic suppression for the same reasons. Then, the intermissions between any two successive PBs provide the time necessary for the recurrent synaptic excitation to regain its excitability. During these burst-free intermissions, single AP spikes prevail and the system recovers its excitability with the outnumbering excitatory synaptic connections over the inhibitory connections. The balance between the suppression and the recovery process sustains the spontaneous PB dynamics.

An extrinsic, stimulation pulse (for example, 60 pA amplitude, 1 ms duration given to a randomly selected group of 20 neurons) evokes an immediate PB, during which almost all neurons fire simultaneously. Each evoked PB has a firing rate profile which is quite skewed toward the onset of excitation. Then, the skewness becomes more conspicuous as the stimulation repeats as shown in the right frame of Fig. 1e. The ‘front heavy’ spike firing rate profile of the evoked PB accelerates the overall suppression of the synaptic connections and breaks the delicate balance between the suppression and the natural recovery process. After being exposed to a long series of external stimuli, only a small set of neurons that receive the stimulation directly and their immediate neighbors would be affected by the stimulation. By then, the perturbation cannot be relayed to the whole neuronal population. The increasing overall inhibition under a repeating stimulation is rather evident in three raster plots of Fig. 1c, in which recurrent burst activity following the perturbation (red vertical line) gets weakened and dies out gradually. In other words, the system is overwhelmed even by a sparse (3 s interval) pacing given only to 5% of the total neuronal population.

### Novelty of Δ*t* paired-pulse stimulation

**Figure 2:**
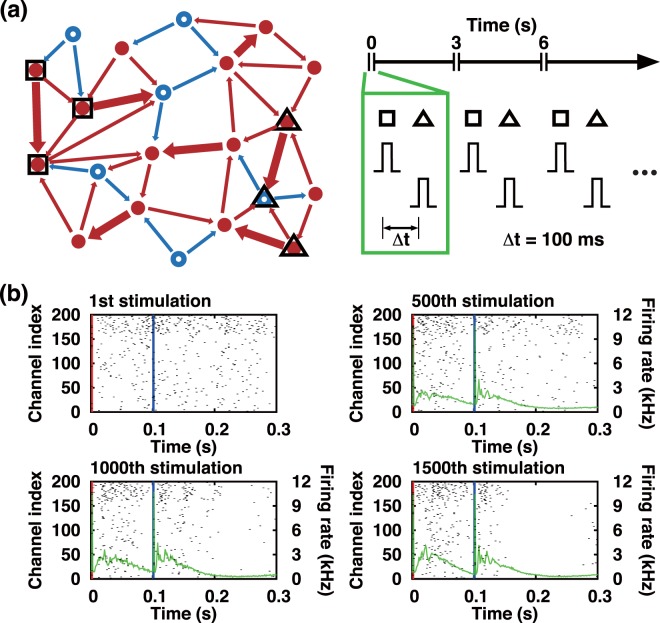
A series of Δ*t* paired-pulse stimuli and its impact on PB dynamics. (**a**) Two groups (squares and triangles) of neurons selected for a Δ*t* paired-pulse stimulation are marked; shown in the right-hand side is an illustration of Δ*t* = 100 ms paired-pulse stimulation sequence [‘square group’ (‘triangular group’) receives the 1st (2nd) pulse for each paired-pulse stimulation], (**b**) Raster plots of APs during and immediately after the labeled Δ*t* paired-pulse stimulation. The superimposed green solid lines are the mean AP firing rate of the evoked PBs taken over 500 successive stimuli (eg., from the 1st to 500th, 501th to 1000th, and 1001th to 1500th). The red and blue vertical lines mark the times of the first and second pulses of a Δ*t* paired-pulse stimulation.

In order to process and encode complex input information (eg., high-frequency spatiotemporal sensory signal), a neural network needs to utilize its whole network connectivity and be away from the network suppression like the one discussed in Fig. 1. For the PB generating Izhikevich neuronal model network, a high frequency (eg. 10~50 Hz) input information can be encoded without causing a complete network suppression, for example, as in the following Δ*t* stimulation protocol, in which two selected groups of neurons are stimulated by a pair of pulses with a relative (small) time delay of Δ*t* (see Fig. 2a, right frame) with a typical range of 50~120 ms.

Essentially, the Δ*t* paired-pulse stimulation protocol reinforces the STDP rule simultaneously to two subpopulations of a large coupled neuronal network having many random recurrent connections. Having two evoked PBs in a short duration of time can result in a rather unexpected, interesting consequence. Two PBs together back-to-back with a small delay can make the system much less suppressed (when compared with the case of a single evoked PB) as illustrated in Fig. 2b: Almost all APs evoked by the first pulse are advanced in time than the APs generated by the second pulse for each paired-pulse stimulation; thus, there would be sufficiently many pre- and post-synaptic pair events that yield a synaptic potentiation over depression. That is so, even when the conduction time delays of 0~20 ms are taken into account. Now, the synaptic potentiation can compete with the depression to find a balance. Therefore, even with the increasing number of Δ*t* stimuli each of the AP firing rate profiles of the two paired PBs does not go into a depression but gradually stabilizes into a non-trivial steady-state (the solid purple lines in Fig. 2b) which is not much different from that of the spontaneously generated burst shown in the first frame of Fig. 1e. In other words, the Δ*t* stimulation (a simple spatiotemporal input pattern having a high-frequency component) could be successfully encoded into the whole network. After all, Δ*t* paired-pulses can be far less suppressing than single pulses.

**Figure 3:**
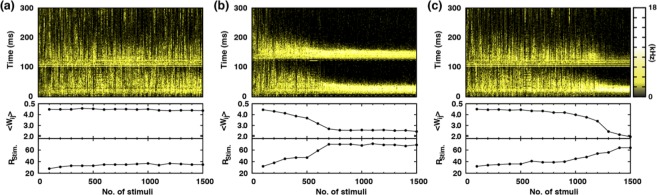
Temporal evolution of AP firing rate and network property following a very long sequence of Δ*t* paired-pulse stimuli. (**a**) Δ*t* = 100 ms, (**b**) Δ*t* = 120 ms, (c) Δ*t* = 100 ms [the same as in (**a**) but with different group of neurons used for the Δ*t* training]. The black-yellow color-maps in the top row depict the AP firing rate following each Δ*t* paired-pulse that is delivered at every 3 s interval, which is indexed along the x-axis. Along the y-axis ‘0’ represents the time of the first pulse being delivered. The second and third row plot the mean synaptic weight 〈*W*_*ij*_〉 and *R*_*Stim*_ as a function of the stimulation sequence index, respectively. *R*_*Stim*_ is the number of APs in a 5 ms time window about the global maximum of the firing rate profile of each PB; so, it measures the simultaneity of the APs comprising each PB.

The purpose of the Δ*t* stimulation is to *train* a PB generating network without completely suppressing its network connectivity; for that matter, a sufficient temporal overlap between the first evoked PB and the following second PB is crucial and we discuss that with Fig. 3. The heat maps in the top row of Fig. 3 quantify the AP firing activity while the system is externally driven by a periodic (period 3 s) sequence of Δ*t* paired-pulse stimuli. For Fig. 3a, Δ*t* = 100 ms is used. During the entire 1,500 rounds of Δ*t* = 100 ms paired-pulse stimulation, except only for the initial transient period of ~600 s (200 stimuli), the pairs of PBs being generated by the stimuli seem quite stable, judging not only from the AP firing rate heat map of Fig. 3a (top frame) (also, see the AP firing rate profiles of Fig. 2b) but also from 〈*W*_*ij*_〉 and *R*_*stim*_, which are the mean value of all synaptic weights and a measure of the simultaneity of the neural spikes associated with each PB, respectively (see middle and bottom frames of Fig. 3a).

### The significance of Δ*t* in the paired-pulse stimulation protocol

**Figure 4:**
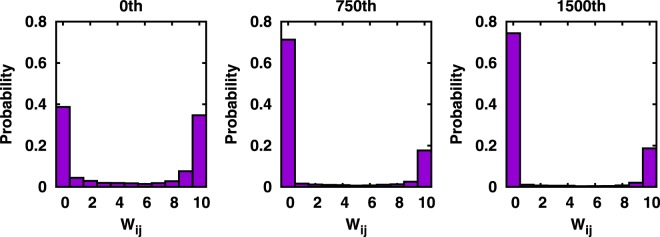
Time evolution of the distribution function of synaptic weights subject to a sequence of Δ*t* paired pulses.

The consequence of the perturbation can be quite different with a larger value of time delay between two stimulating pulses, for example, Δ*t* = 120 ms (see Fig. 3b): Now, the system becomes severely depressed as the profiles of two evoked PBs get quickly separated in time and their APs loose STDP mediated interactions. The increasing separation is quite clear in the color activity map of Fig. 3b. Notably, for this case, 〈*W*_*ij*_〉 decreases while *R*_*stim*_ rises sharply as the number of stimuli increases. A large value of *R*_*stim*_ means that there are too many simultaneous AP firings that would cause an overall synaptic depression. Again, for such a case the conduction delays (*d* = *τ*_*ij*_) are a major factor determining the (suppressing) action of the STDP function shown in Fig. 1b. Eventually, all the synaptic weights, except for those of output connections emanating from the neurons that directly receive the extrinsic perturbation, go to zero. Figure 4 quantifies the evolution of probability distribution function of *W*_*ij*_ for such a case. We view that $\langle {W}_{ij}\rangle \,\lesssim \,2$ represents a nearly complete network suppression. In fact, any paired-pulse stimulation with $\Delta t\gtrsim 120$ ms is observed to undergo a complete suppression.

If the value of Δ*t* gets too small (eg. 20 ms), the two evoked PBs practically overlap each other to form a (roughly twice) densely packed single-hump PB that would give a more severe depression to the network connectivity than a single pulse stimulation would induce. After all, there should be an appropriate range of Δ*t* values, with which one can train the network without causing a complete depression. For the selected set of system parameter values that we have used in this work, the ‘non-depressive Δ*t* range’ is approximately 30~120 ms: Only for this range 〈*W*_*ij*_〉 is greater than 4 and it decreases sharply to ~2 below 30 ms and beyond 120 ms.

The degree of stimulation-driven depression is, of course, a function of the number of stimuli received by the system as shown in Fig. 3b: The depression sets in gradually and saturates around the 700th stimulation. More importantly, the degree of depression also depends on the group of selected neurons that receive the stimulation. For example, the result shown in Fig. 3c is obtained with the same Δ*t* = 100 ms that was used for Fig. 3a, but with a different group of neurons being stimulated. For the case of Fig. 3c, the system gets depressed rather slowly at the beginning but accelerates quickly around 1000th stimulation to a complete depression. After all, these features (the dependence on the value of Δ*t*, the group of neurons receiving the stimulation, and the number of stimuli) are exactly what one would want for encoding spatiotemporal information into the network.

### Accessing the excitability of Δ*t*-trained networks by a probing pulse stimulation

**Figure 5:**
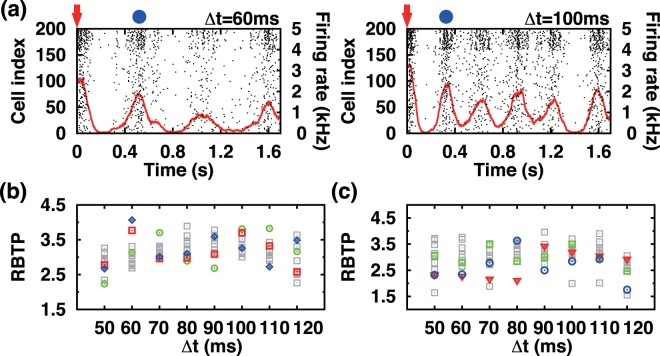
Significance of Δ*t* value in Δ*t* paired-pulse stimulation. (**a**) Two exemplary raster plots showing an almost periodic sequence of recurrent bursts following a probing pulse stimulation, which is marked by a red arrow [after Δ*t* = 60 ms training (left) or Δ*t* = 100 ms training (right)]. (**b**) shows RBTP vs. Δ*t* for 10 different initial *W*_*ij*_ values just before the training for each value of Δ*t* (only three randomly chosen *W*_*ij*_ initial states are color labeled to illustrate the variability of RBTP following the initial state of *W*_*ij*_, (**c**) shows RBTP vs. Δ*t* for 10 different groups of neurons used for the Δ*t* stimulation [as in (**b**), only three randomly chosen cases are color labeled]. The same initial condition (i. e., *W*_*ij*_ values) is used for the case of (**c**), while the same groups of cells are used for the training in (**b**). In (**a**), the red solid lines plot the rate of APs and the peak position of the first recurrent burst is marked by a blue dot. The value of RBTP plotted in (**b**) is based on 100 peak positions (blue dots) generated by 100 probing pulses while the values of *W*_*ij*_ are kept after the Δ*t* paired-pulse training.

A useful information about the dynamic state of a Δ*t*-trained network can be extracted by delivering a probing pulse. A probing stimulation is a single pulse (60 pA amplitude, 1 ms duration) stimulation simultaneously given to a set of (randomly) selected 20 neurons. The raster plot of Fig. 5a (left frame) shows a typical response of the system to a probing pulse stimulation, which immediately follows a Δ*t* training session. The almost periodic sequence of recurrent PBs (with a period ~0.5 s) can be viewed as a reminiscence of the noisy subthreshold PB oscillator that was discussed in ref.^13^. The recurrent PBs shown in Fig. 5a (left frame) are produced by a network which is trained with 300 Δ*t* = 60 ms paired pulses, while the ones shown in Fig. 5a (right frame) are for 300 Δ*t* = 100 ms paired pulses. Clearly, the recurrent PBs in the left frame of Fig. 5a are more dispersed than those in the right frame of Fig. 5a.

Importantly, the timing precision RBTP ≡ <*T*>/*σ*(*T*) (or RBTP = 1/coefficient of variation of *T*) of the recurrent bursts following a probing pulse stimulation depends on the value of Δ*t*. Here, <*T*> and *σ*(*T*) refer to the mean and standard deviation of the time intervals between the probing pulse stimulation (marked by a red arrow in Fig. 5a) and the first recurrent burst (marked by blue dot in Fig. 5a) measured over 100 different trials given at an interval of 10 seconds (while the values of *W*_*ij*_ are fixed after the completion of each Δ*t* training). Figure 5b plots RBTP vs. Δ*t* for ten different initial network states (i. e., different initial *W*_*ij*_). Clearly, the value of RBTP depends not only on the value of Δ*t* but also on the initial network connectivity structure, the one just before the training pulses are begun to be delivered. In other words, the encoded memory state (accessed by the measure of RBTP) depends on the past history of the network before the training process starts. The dynamic network evolves autonomously in time, and practically the high dimensional system will never be in the same dynamics state when the stimulation is being delivered. In other words, a new input information gets encoded on the top of what is currently being stored in the network. Furthermore, as expected the value of RBTP also depends on the specific composition of the two groups of neurons receiving the training stimuli directly as well as on the value of Δ*t* used for a priori training session (see Fig. 5c). There seems to be no simple functional relationship between the value of RBTP and Δ*t* for the range of Δ*t*, for which the system does not go into a complete inhibition.

### Recurrent burst timing precision (RBTP) versus other measures of the network

**Figure 6:**
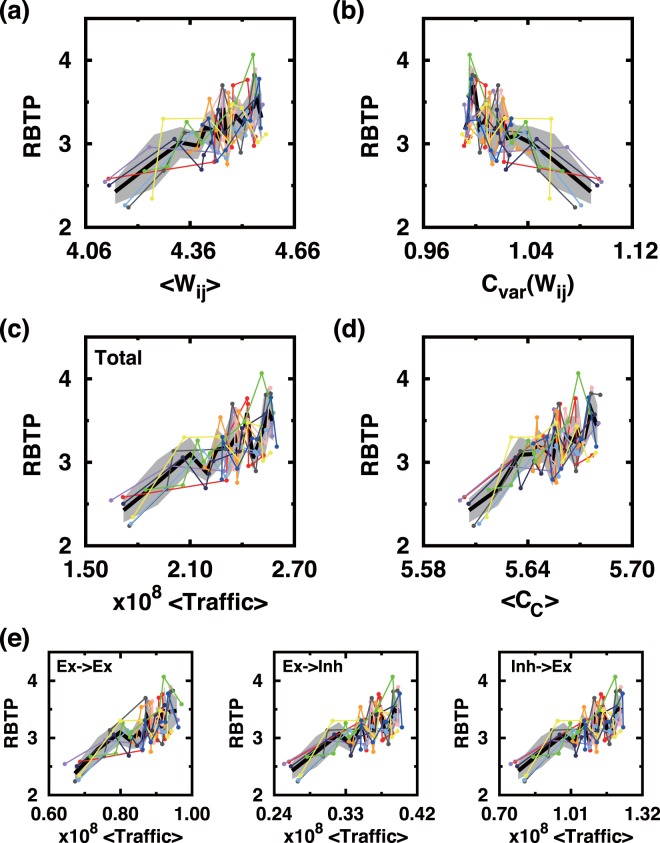
RBTP versus other measures characterizing the network connectivity. (**a**) The mean of synaptic weights *W*_*ij*_, (**b**) the covariance of synaptic weights *W*_*ij*_, (**c**) the mean of synaptic transmissions (〈Traffic〉), and **(e**) the mean of closeness centralities (*C*_*c*_). (**d**) RBTP vs 〈Traffic〉 for three different sub-types of synaptic connections. Ten different colors represent 10 different initial states and eight different dots along each line correspond to 8 different Δ*t* values (equally spaced from 50 to 120 ms). For the exact definition of synaptic traffic and closeness centrality, see the text. The thick black lines connect local averages (over 5 neighboring data points) and the background gray shades represents the corresponding standard deviation.

The level of individual neural spiking activity can be considered as a measure of the overall excitability that is relevant for the emergence of a population burst. The mean synaptic strength 〈*W*_*ij*_〉 is a major factor affecting the spiking activity, and on average it is almost a linear function of RBTP: Shown in Fig. 6a are noisy, yet, almost linear functional relationships between 〈*W*_*ij*_〉 and RBTP for 10 different initial states (marked by different colors) and 8 different Δ*t* values (dots connected by a line). RBTP also depends on the uniformity of *W*_*ij*_: The larger the coefficient of variation C_*var*_ of *W*_*ij*_ is, the smaller RBTP is (see Fig. 6b). In other words, the more homogeneous the network connection weights are, the more reliable and accurate the recurrent PBs are when they are induced by a probing pulse stimulation. Also, RBTP is a linearly increasing function of the actual amount of synaptic transmission (traffic), which is defined by the sum of all weighted synaptic inputs triggered by APs during the total Δ*t* training period of 900 s (see Fig. 6c). The total synaptic transmission can be further divided into three different categories, namely, the transmission from excitatory neurons to another excitatory neurons, that from excitatory neurons to inhibitory neurons, and that from inhibitory neurons to excitatory neurons. In all three cases, RBTP is found to be a linearly increasing function of synaptic transmission (see Fig. 6d).

Another important factor that characterizes the connectivity of a functional network is the closeness centrality *C*_*C*_, which measures the degree of how near each neuron is to all other neurons or how well neural information spreads on the network. *C*_*c*_ is computed by the following procedure. First of all,

$${\cos }{{t}}_{ij}=\frac{1}{{W}_{ij}}$$

is computed for all connected pairs from *i*th neuron to *j*th neuron (only *W*_*ij*_ > 0 is considered). Then, the weighted distance between *m*th neuron and *n*th neuron (*dist*_*mn*_) is defined to be the shortest path *P*_*mn*_ connecting the *m*th neuron to the *n*th neuron:

$$dis{t}_{mn}=\sum _{i,j\in {P}_{mn}}\frac{1}{{{cost}}_{ij}}.$$

Finally,

$${C}_{C}(m)=\frac{|{N}_{m}|-1}{{\sum }_{n\in N}dis{t}_{mn}},$$

where *N*_*m*_ is the number of neighbors of the neuron indexed by *m*. The program CytoNCA^37^ is used to calculate *C*_*C*_. Figure 6d plots RBTP vs. 〈*C*_*C*_〉. As expected a well-connected network with a larger value of 〈*C*_*C*_〉 gives a better precision in the timing of the recurrent burst. Thus, the ‘overall excitability’ of the PB generating neural system surely depends on the degree of network connectivity as well as the network homogeneity and 〈*W*_*ij*_〉.

**Figure 7:**
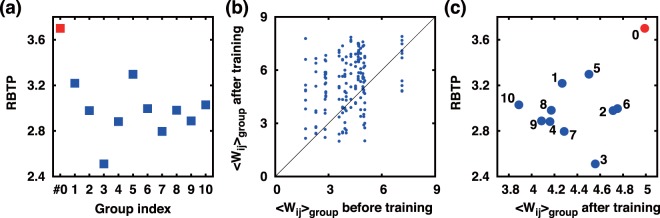
The dependence of RBTP on the groups of neurons that receive a *probing* pulse (**a**) RBTP vs. group used for probing pulse stimulation (#0 red is for the same group of neurons that were used for Δ*t* training beforehand, all others are just randomly selected groups of neurons), (**b**) The overall increase in the mean synaptic weight of neurons which were chosen to receive the Δ*t* = 100 ms training pulses, and (**c**) RBTP vs. 〈*W*_*ij*_〉_g*roup after training*_. Note in (**a**) that RBTPs for 10 randomly chosen groups of neurons all are not as good as that of the first recurrent burst generated by a probing pulse delivered to the group of neurons that were used for the prior Δ*t* training.

The value of RBTP also strongly depends on the location where within the network the probing pulse stimulation is delivered, as the network connection morphology matters for the value of RBTP. Naturally, the probing pulse delivered to the very group of neurons (labeled by group index #0 in Fig. 7a), which was used for the Δ*t* training, yields the largest RBTP over 10 other trials made with other groups of neurons selected for the probing stimulation (see Fig. 7a). As explained earlier, during the time course of a Δ*t* training, the efferent synaptic weights of the neurons that receive the training pulses would be generally enhanced (from 〈*W*_*ij*_〉_g*roup before training*_ to 〈*W*_*ij*_〉_g*roup after training*_), and Fig. 7b well confirms that: The mean synaptic weight of those neurons used for the Δ*t* training has significantly increased after the training process. After all, the mean synaptic weight is the largest (~5) for the group of neurons that received the training pulses directly and RBTP is the largest when the same group of cells are stimulated by a probing pulse: The case is marked by a red dot and index #0 in Fig. 7c. In other words, with an appropriate Δ*t* training some strong pathways are established connecting the group of neurons, which receive the Δ*t* stimuli directly, to other neighboring neurons. The input information is impregnated into the network weight morphology forming a memory.

## Summary and Discussion

Essentially all STDP rules, developed so far, are for a pair of neurons: The rules consider the time difference(s) between two (or more) spikes, for example, one for the pre-synaptic neuron and the other for the post-synaptic neuron, and modifies the strength of the synapse connecting them as a nonlinear function of the time difference. Understanding the outcome of this rule applied to not just a pair but many neurons forming a well-connected network is far from trivial due to the intrinsically polysynaptic nature of the circuits having plastic and recurrent connections. Interesting issues can emerge 91) if such a system autonomously generate population bursts with a more or less well-defined time interval and 92) if it is perturbed by a periodic sequence of pulses. Self-sustained PB activity is sustained with a delicate balance of excitation and inhibition, as discussed in ref.^38^. When the PB generating network is stimulated at a high frequency, it will become easily depressed and fragmented. On the other hand, a temporally sparse stimulation will neither affect the autonomous network bursting activity in any meaningful way nor be able to form any input-specific synaptic structural adaptation in the network. The Δ*t* paired-pulse stimulation protocol that we developed and implemented both in the earlier experiments^29^ and the current model study is in a way a compromise between these two extremes and works in a regime causing significant changes to the network connectivity, yet, avoiding a complete depression. Most importantly, the Δ*t* stimulation protocol includes a high-frequency component (i.e., the small value of Δ*t*), which is relevant for the STDP rule, thereby enforcing a non-trivial interplay between the network plasticity and the external information.

In addition to the time delay Δ*t*, which is associated the extrinsic paired-pulse perturbation, our model has also incorporated uniformly distributed conduction time delays *τ*_*ij*_: They were added to the time difference of spiking of the coupled neurons when the STDP function was evaluated. The time delays *τ*_*ij*_ were, more precisely speaking, axonal conduction time delays as versus to dendritic time delays. According to a series of recent phase model (for weakly pulse-coupled oscillators) studies by Madadi Asl *et al*.^39,40^, the axonal and dendritic conduction delays both could be delicate parameters so that, for example, their different balances could bring quite a few different network connectivity patterns, including recurrent bidirectional couplings. In addition, their recurrent network model could also support multistability that were rendered visible with different initial distribution of the synaptic strengths. Knowing that in our model system the value of RBTP (after a Δ*t* stimulation) has fluctuated significantly depending on the initial landscapes of *W*_*ij*_ as shown in Fig. 5b, it is tempting to speculate that the fluctuation could be originating from the multistability of our (unperturbed) network model. So, in the future it will be interesting to check the possibility of multistability and explore the role of conduction time delays (eg. non-uniform distributions of axonal and dendritic time delays and their different balances) in a systematic way.

Earlier, we claimed that the recurrent burst timing precision RBTP is a good readout measure of the overall excitability relevant for the emergence of a population burst^29^. In this work, we demonstrated that not only the mean synaptic weight 〈*W*_*ij*_〉 but also the network connectivity, as measured by *C*_*var*_, and the homogeneity, as measured by 〈Traffic〉 and 〈*C*_*C*_〉, are an important factor as for determining the overall excitability. In addition, we showed that the value of RBTP also relies on the morphology of the trained network: The detailed landscape of synaptic weights matters as for determining RBTP, as the probing pulse is given only to a small subset of neurons. After all, the Δ*t* paired-pulse stimulation conveys an externally imposed spatiotemporal information to the network and forms a memory in the plastic landscape of synaptic weights. At this point, we should indicate that synaptic plasticity is often explored as a form of unsupervised adaption of neural circuits learning the structure of complex sensory inputs^41^. The evaluation of RBTP following the probing pulse stimulations is in a way for retrieving the Δ*t* stimulation-encoded information. However, there is no simple, a priori set, functional relationship between RBTP and the value of Δ*t*, as the initial state of the given network, the number of stimulations being given, and the subgroups of cells receiving the stimulations are also an important factor.

## Conclusion

The modulation of RBTP in cultured networks of cortical neurons by Δ*t* stimulation reported earlier in ref.^29^ was successfully reproduced in computer simulations of a simple neuronal network model in which synaptic weights evolve according to a simple STDP rule as described in^32^. Subsequently, with a series of careful analyses we elucidated the significance and novelty of the Δ*t* paired-pulse stimulation protocol as for an effective way of inducing input-specific structural changes as well as for modifying the overall bursting excitability of neuronal network. Finally, we indicate that the Δ*t* stimulation that we developed may represent one of the most simplest forms of spatiotemporally structured sensory inputs that have a high frequency component. As such, in the future we will explore how the same burst-generating neural network would adapt its synaptic weights in response to more complex spatiotemporally patterned stimuli. Moreover, we can explore the same issues with a more sophisticated STDP rule that includes triple-spike, or even quadruple-spike, nonlinear interaction as in^42–44^ for our future work.

## Data Availability

All numerical data used in this work will be available upon request to the corresponding author.
